# Determinants of adults' intention to vaccinate against pandemic swine flu

**DOI:** 10.1186/1471-2458-11-15

**Published:** 2011-01-06

**Authors:** Lynn B Myers, Robin Goodwin

**Affiliations:** 1 School of Social Sciences, Brunel University, Uxbridge, UK

## Abstract

**Background:**

Vaccination is one of the cornerstones of controlling an influenza pandemic. To optimise vaccination rates in the general population, ways of identifying determinants that influence decisions to have or not to have a vaccination need to be understood. Therefore, this study aimed to predict intention to have a swine influenza vaccination in an adult population in the UK. An extension of the Theory of Planned Behaviour provided the theoretical framework for the study.

**Methods:**

Three hundred and sixty two adults from the UK, who were not in vaccination priority groups, completed either an online (n = 306) or pen and paper (n = 56) questionnaire. Data were collected from 30th October 2009, just after swine flu vaccination became available in the UK, and concluded on 31st December 2009. The main outcome of interest was future swine flu vaccination intentions.

**Results:**

The extended Theory of Planned Behaviour predicted 60% of adults' intention to have a swine flu vaccination with attitude, subjective norm, perceived control, anticipating feelings of regret (the impact of missing a vaccination opportunity), intention to have a seasonal vaccine this year, one perceived barrier: "I cannot be bothered to get a swine flu vaccination" and two perceived benefits: "vaccination decreases my chance of getting swine flu or its complications" and "if I get vaccinated for swine flu, I will decrease the frequency of having to consult my doctor," being significant predictors of intention. Black British were less likely to intend to have a vaccination compared to Asian or White respondents.

**Conclusions:**

Theoretical frameworks which identify determinants that influence decisions to have a pandemic influenza vaccination are useful. The implications of this research are discussed with a view to maximising any future pandemic influenza vaccination uptake using theoretically-driven applications.

## Background

In April 2009, a new strain of influenza virus H1N1 (swine) flu, unrelated to human seasonal influenza viruses, was reported in Mexico, and spread rapidly around the world. The World Health Organisation (2009) declared swine flu a global pandemic on 11 June 2009, by which time it was estimated that 24,000 had been infected, and 143 had died [1].

Soon after identification of this new strain of H1N1, a 2009 vaccination for H1N1 was developed to be administered separately from the seasonal influenza vaccination [2]. In the UK, the Government ordered 90 million doses of swine flu vaccine - enough to vaccinate the entire population, and more per head than any other country in Europe [3]. UK vaccination began on 21 October 2009, with the highest-risk groups, including health professionals, being offered the vaccine first. During the latter stages of 2009, it was intended that after vaccination was offered to the high priority groups, vaccination was going to be available for everybody else [4].

Earlier in the current H1N1 alert, two studies had investigated intention to have a vaccination amongst those seen to be at risk. A survey of health workers in Hong Kong indicated that during pre-pandemic stage 3 only 28.4% intended to accept vaccination with pre-pandemic vaccine, which increased to 34.8% during pre-pandemic stage 5 [5]. An online survey in Italy during August 2009 investigating parents' willingness to have their child vaccinated with H1N1 vaccine reported that only 12.8% of mothers said they would allow their children to be vaccinated and 44.4% remains doubtful [6].

To the authors' knowledge no studies have investigated non-priority group adults' intentions to be vaccinated when the H1N1 vaccine became available. At the time of the current study, it was believed that H1N1 was an extremely serious health threat to everyone [4]. It is important therefore to investigate predictors of intention to have a vaccine among the general population during the 2009 pandemic in order to be able to inform vaccine uptakes in any future pandemics. The current study investigated intention to vaccinate in a non-priority, adult population in the UK.

Social cognition models have been widely used to predict intention and behaviour [7]. The Theory of Planned Behaviour [8] has been applied to an extensive range of health-related behaviours [9], and is probably the most influential and robust social cognition model in predicting and explaining health behaviour [10]. This model suggests that the proximal determinants of behaviour are intention to perform that behaviour and perceived behavioural control (the person's own subjective perception of whether they can perform the behaviour). The predictors of behavioural intention are attitudes to the behaviour (a favourable or unfavourable evaluation of the particular behaviour), subjective norm (perceived social pressure to perform or not perform the behaviour) and perceived behavioural control [8,9]. Perceived behavioural control comprises two separate factors: self-efficacy (a person's perceived confidence and perceived difficulty in performing the behaviour [11] and perceived control (a person's belief about their ability to control the behaviour in question [12].

Despite the success of the Theory of Planned Behaviour in predicting intention and behaviour, new components are continually being added to the model to increase its usefulness. There are very few studies investigating intention to have an influenza vaccination. However, in a study of older adults' intention to have a seasonal influenza vaccination, the addition of anticipated regret substantially increased the predictive value of the Theory of Planned Behaviour [13]. Anticipated regret may be experienced when we realise or imagine that the present situation could have been better if we acted differently [14,15]. In addition, studies have found past behaviour [13,16,17] and knowledge [18,19] to be further predictors of intention and behaviour. Finally, earlier research on swine flu vaccination used the original Health Belief Model to predict intention and behaviour in the USA [20,21]. We therefore also included the four variables used in this model (perceived susceptibility (risk of getting the condition); perceived severity (seriousness of the condition); perceived benefits (positive consequences of adopting the behaviour), and perceived barriers (influences that discourage adoption of the behaviour).

The second aim of this study was to investigate whether intention to have a swine flu vaccination was related to ethnicity. Previous research has suggested that African Americans are less likely to have a seasonal influenza vaccine [22,23]. For example, one such study concluded that this was because African Americans are less likely to initiate medical encounters for the purpose of being vaccinated than Whites [23]. The current study investigated whether there would be a similar pattern for intention to have swine flu vaccination in the UK.

Thirdly, a number of information leaflets about swine flu were produced jointly by the UK Department of Health and by the National Health Services in England, Northern Ireland, Scotland and Wales. "Important information about swine flu," a detailed (11 page) document [24] was delivered to every household in the UK during May 2009, as well as being available online. This leaflet included information about what swine flu is and how it could spread; the UK Government's response in preparation for a wider pandemic; protective actions to protect against swine flu (e.g. carry tissues, binning the tissues after one use); advice about face masks (that these were not effective); what to do if you think you had flu symptoms; and how to keep up-to-date with the latest information about swine flu. It also included a telephone number for further advice. In addition, there were also a number of Department of Health television adverts, posters and information in newspapers.

In October 2009, a detailed (12 page) information leaflet for swine flu vaccination ("Swine flu vaccination: what you need to know") described the nature of swine flu (e.g. a respiratory disease, the severity of the threat), gave information about the swine flu vaccine (the two brands of vaccine, the differences between the swine flu vaccination and the seasonal flu vaccination), and identified priority groups (e.g. adults and children over six months or age with a long-term health condition, pregnant women, people living in the same house as someone with a compromised immune system, health and social care staff who have close contact with the above groups). In addition, the leaflet described who could not have the vaccination, the use of Celvapan (rather than Pandemrix) for those with egg allergies, the potential safety of the vaccine and possible side effects, and the need for the vaccine amongst those who already have had swine flu. However, this leaflet was not delivered to households [25]. Therefore, the current study also investigated knowledge about swine flu vaccination.

## Methods

### Participants

This was part of an international study on swine flu vaccination which investigated intention to have a swine to vaccination in the UK, China, North America, Turkey and Hungary. Exclusion criteria for this paper were: non UK residents, health care workers and other vaccination priority groups [4].

### Procedure

Following ethical approval from Brunel University, UK, the questionnaire was linked to the website http://www.vaccinequestionnaire.com, which was established for these purposes. There was also a paper version. Data were collected via the snowballing methodology. The website link was also pasted onto a variety of general, networking websites (e.g. I love London), advertised through the pages of social networking sites (e.g. Facebook) and announced on the Brunel University intranet. In addition, colleagues, neighbours and friends were contacted by e-mail and in person with details of the study and were also asked to advertise the survey. Contacts who asked for a paper version rather than the online version were given copies of the questionnaire. Data were collected from 30th October 2009, just after swine flu vaccination became available in the UK, and concluded on 31st December 2009.

### Measures

These were components of the extended Theory of Planned Behaviour (intention, attitude, subjective norm, self-efficacy, perceived control, anticipated regret, past-related behaviour, future-related behaviour, knowledge), Health Belief Model variables (perceived susceptibility, perceived severity, perceived benefits, perceived barriers), and demographic variables (age, gender, employment status and ethnicity). Participants were also asked whether they were high priority for swine flu vaccination and the reason(s) for this.

### Components of an extended Theory of Planned Behaviour

All items were measured on a 7 point Likert scale, unless otherwise stated.

***Intention*** was measured with a single item: "Do you intend to have a vaccination for swine flu?" with (1) anchored at "definitely will not" and (7) anchored at "definitely will. A high score indicated a more positive intention to have a vaccination.

***Attitude ***was measured using one statement: "If I were to have a vaccination for swine flu it would be," followed by a series of 6 semantic differential scales:- wise-foolish, worthless-valuable (reverse score), beneficial-harmful, satisfactory-unsatisfactory, bad-good (reverse score), positive-negative. Cronbach alpha for these items was 0.97 and so the mean of the 6 items was taken as an overall measure of attitude, with lower scores indicating a more positive attitude towards vaccination.

***Subjective norm ***was measured by 5 items. Three items were worded as follows: "People who are (a) important to me (b) my family (c) my friends would" approve [anchored at 1] - disapprove [anchored at 7]... "of my having a swine flu vaccination" (from approve to disapprove), plus two items were worded as follows: "(d) I feel under social pressure to have a swine flu vaccination" and "(e) people who are important to me influence my decision to have a swine flu vaccination," with (1) anchored at "strongly agree" and (7) anchored at "strongly disagree." Cronbach alpha for these items was 0.79 and so the mean of the 5 items was taken as an overall measure, of with lower scores indicating high subjective norm.

***Self-efficacy ***was measured by 3 items: (a) "For me to have a swine flu vaccination would be difficult - easy, with (1) anchored at " difficult" and (7) anchored at "easy;" (b)" if I wanted to I could easily have a swine flu vaccination" [reverse score] with (1) anchored at "extremely true" and (7) anchored at "extremely untrue" and (c) "how certain are you that you could have a swine vaccination?" [reverse score] with (1) anchored at "not at all certain" and (7) anchored at "very certain." Cronbach alpha for these items was 0.89 and so the mean of the 3 items was taken as an overall measure of self-efficacy, with lower scores indicating high self-efficacy.

***Perceived control ***was measured by 3 items: (a) "The number of events outside my control which would prevent me from having a swine flu vaccination are" [reverse score]: numerous-very few, with (1) anchored at "numerous" and (7) anchored at "very few;" (b)" it is mostly up to me whether or not I have a swine to vaccination" [reverse score], with one end of the scale, "strongly disagree" anchored at (1) and the other end of the scale "strongly agree" anchored at (7), and (c) "how much personal control do you have over whether you do or do not have a swine flu vaccination? [reverse score]. One end of the scale, "very little control" was anchored at (1) and the other end of the scale "total control" was anchored at (7). Cronbach alpha for these items was 0.79 and so the mean of the 3 items was taken as an overall measure of perceived control, with lower scores indicating high perceived control.

***Anticipated Regret*** was measured with a single item: "If I did not have a swine flu vaccination, I would later wish I had", with (1) anchored at "strongly agree" and (7) anchored at "strongly disagree." A low score indicated higher anticipated regret.

***Past and future- related behaviours ***concerned normal seasonal flu vaccination. Past- related behaviour was measured with an individual item: "Last year, did you have a vaccination for ordinary seasonal flu?" Future- related behaviour was measured with an individual item: "This year, do you intend to have a vaccination for ordinary seasonal flu?" These 2 questions were rated as "yes", "no" or "don't know". No participant answered "don't know" for past-related behaviour and only 8 answered "don't know" for future-related behaviour, so the "don't know" option was combined with "no".

***Knowledge ***consisted of 7 questions taken from the UK swine flu vaccination leaflet [25]. These were: (1) "There is much more risk of side-effects from swine flu vaccination vs. the regular seasonal flu vaccination," (2) "it is safe for pregnant women to have swine flu vaccination," (3) "swine flu vaccination will also protect you against seasonal flu," (4) "most reactions to swine flu vaccination are mild," (5) "swine flu vaccination can cause swine flu" and (6) "most reactions to swine flu vaccination do not last longer than two days." There was also a question about vaccinations in general. "All vaccinations can cause side-effects." Ratings were "yes", "no" or "don't know." For questions 1 and 5 the correct answers are "no" and for questions 2, 3, 4, 6 and 7 the correct answers are "yes." An overall knowledge score was obtained from the 6 swine flu questions, collapsing the wrong answer with the "don't know" option for each question and summing responses.

***Health Belief Model Variables***. These were all measured with 3 items on 7 point Likert scales with (1) anchored at "strongly agree" and (7) anchored at "strongly disagree."

***Perceived Susceptibility of contracting swine flu ***was measured by: (a) My chance of getting swine flu in the next few months is great," (b) "I am worried about the likelihood of getting swine flu in the near future," and (c) "getting swine flu is currently a possibility for me." Cronbach alpha for items on the scale was 0.75, so the mean of the 3 items was taken as an overall measure of perceived susceptibility, with a low score indicating high perceived susceptibility.

***Perceived severity ***of swine flu was measured by: (a) "Complications from swine flu are serious," (b) I will be very sick if I get swine flu," and (c) "I am afraid of getting swine flu." Cronbach alpha for items on the scale was 0.76, so the mean of the 3 items taken as an overall measure of perceived severity, with a low score indicating high perceived severity.

***Perceived benefits of swine flu vaccination ***was measured by: (a) "Vaccination is a good idea because I feel less worried about catching swine flu;" (b) "vaccination decreases my chance of getting swine flu or its complications," and (c) "if I get vaccinated for swine flu, I will decrease the frequency of having to consult my doctor." As these items measured separate benefits [26] they were analysed separately and no internal reliability analysis was conducted. Low scores indicating high perceived benefits.

***Perceived barriers of swine flu vaccination ***were measured by: (a) "The side-effects of swine flu vaccination interfere with my usual activities," (b) "I am scared of needles," and (c) "I cannot be bothered to get a swine flu vaccination." As these items measured separate obstacles [26,27] they were analysed separately and no internal reliability analysis was conducted. Low scores indicating high perceived barriers.

### Demographic Details

Participants were asked their age, gender (coded as: 1 = female, 2 = male), employment status (coded as: 1 = employed, 2 = unemployed) and ethnicity. Ethnicity consisted of 12 categories of ethnic origin based on the current UK Census [28].

They were also asked: "are you in a high priority group for vaccination?" This question was rated as "yes," "no" or "don't know." If the respondent answered "yes," they were then asked "why? This was in a free response format which was later categorised by the investigators. There were 5 high priority groups for vaccination [4,25]. These were: (1) People over 6 months in the seasonal flu vaccine at-risk groups (chronic lung disease, chronic heart disease, chronic kidney disease, chronic liver disease, chronic neurological disease, diabetes and people who are immunosuppressed), (2) all pregnant women, (3) people who live with someone whose immune system is compromised (for example, people with cancer or HIV/AIDS), (4) young children aged over 6 months and under 5 years, and (5) frontline health and social care workers.

### Statistical Methods

Analyses were performed using SPSS 15.0 (SPSS, Inc, Chicago, Illinois, USA). Internal consistency reliability among the theoretical constructs was assessed using Cronbach alpha. Bivariate associations were assessed using Pearson's correlation. Differences in ethnicity and employment status were explored using one way analysis of variance and, when appropriate, with SNK post hoc tests with significance levels at p < .05. Some gender differences were explored with Chi Square statistics. Linear hierarchical multiple regression analysis was used to explore which variables predicted intention. Demographic variables (gender, age, employment, whether internet or paper versions of survey) were entered in Block 1, Theory of Planned Behaviour variables (attitude, subjective norm, self-efficacy, perceived control) were entered in Block 2, Extended Theory of Planned Behaviour variables (anticipated regret, knowledge, past-related behaviour, future-related behaviour) were entered into Block 3 and Health Belief Model variables (perceived susceptibility, perceived severity, perceived benefits, perceived barriers) were entered in Block 4. The effect of the independent variables were expressed in terms of standardized regression coefficients (betas). The fit of the model was reported in terms of the adjusted squared multiple correlation coefficient (adjusted R^2^).

## Results

Results were obtained from 362 participants, with 325 participants reporting their age and gender (mean age 31.2, SD 13.37, 62% female). Fifty six participants completed a paper version and 306 completed the questionnaire online. There were no significant differences in age, gender and whether employed between the data collected by internet or the paper version.

### Knowledge

Knowledge was not particularly good. Fifty five point nine percent of participants recognised that swine flu vaccination does not protect them against seasonal flu, and swine flu vaccination cannot cause swine flu was correctly identified by 53%. A slightly lower number, 43%, acknowledged that most reactions to swine flu vaccination are mild. Three items were answered correctly by far fewer participants: (It is safe for pregnant women to have swine flu vaccination (correctly identified by 32%), that most reactions to swine flu vaccination do not last longer than two days (correctly identified by 29.1%) and that there are no more risk of side-effects from swine flu vaccination vs. the regular seasonal flu vaccination (correctly identified by 16.2%). However, there was better understanding about vaccinations per se, as 82.1% acknowledged that all vaccinations can cause side effects.

### Ethnicity

One hundred and twenty six participants reported their ethnicity. Those reporting their ethnicity were significantly older than those that did not (F (1, 361) = 12.90, p < .001), although there were no significant differences in gender and whether employed. Due to low numbers in each cell, the 12 categories of ethnic origin were reduced. Those answering "other" (N = 5) were omitted from further analysis. This resulted in 121 participants. Black or Black British - Caribbean/Black or Black British - African/Black or Black British - Other, were combined into a single variable "Black" (N = 20). Asian or British Asian - Indian/Asian or British Asian - Pakistani/Asian or British Asian - Bangladeshi/Chinese were combined into a single variable "Asian" (N = 29). White" (N = 72) was a combination of White - British/White - Irish/White - Other. One way analysis of variance (ANOVA) indicated a main effect of Ethnicity (F (2, 118) = 4.40, p < 01). SNK post hoc test indicated that Black participants were significantly less likely to intend to have the vaccination vs. Asian and White participants (Black, mean = 1.95, SD = 0.89; Asian, mean = 3.34, SD = 1.80; White, 3.03, SD = 1.80). Age was not significantly different among the 3 groups F (2, 118) = 0.39, p = 0.57), nor was gender (χ^2 ^= 2.08, df = 2, p = 0.37).

### Intention to have a swine flu vaccination

Intention to have a swine flu vaccination was fairly low (mean 2.93, standard deviation = 1.89). Intention was significantly correlated with attitude, subjective norm, perceived control, anticipated regret, perceived susceptibility, perceived severity, all 3 perceived benefits: "vaccination is a good idea because I feel less worried about catching swine flu," "vaccination decreases my chance of getting swine flu or its complications," and "if I get vaccinated for swine flu, I will decrease the frequency of having to consult my doctor, and 2 of the perceived barriers "the side-effects of swine flu vaccination interfere with my usual activities" and "I cannot be bothered to get a swine flu vaccination." (r = -0.68, r = -0.50, r = -0.23, r = -0.63, r = -0.39, r = -0.26, r = -0.61, r = -0.39, r = -0.23, r = -0.19, p <.01, r = -0.22, respectively. Unless stated, the correlations were significant at p < .001). This means that high intention of having a swine flu vaccination was associated with a positive attitude, high perceived control, high perceived susceptibility, high perceived severity, high scores on all of the three perceived benefits items, low scores on the belief that the side-effects of the vaccination would interfere with usual activities and on not being bothered to get swine flu vaccination. Self-efficacy and the perceived barrier item "I am scared of needles" were not significantly correlated with intention (r = 0.05, p = 0.35, r = 0.02, p = 0 67, respectively). ANOVA indicated that employed people were significantly less likely to intend to have a swine flu vaccination (F (1, 323) = 5.80, p < .05) although there were no significant gender differences (F (1, 323) = 2.33, p = 0.24). Age was significantly positively correlated with intention (r = 0.20, p < .001).

To investigate determinants of intention, a hierarchical multiple regression was performed using intention as the dependent variable (see Table 1).

**Table 1:** Hierarchical multiple regression analysis of factors associated with intention to receive 2009 H1N1 vaccination.

Variables	**Adjusted R**^**2**^	Beta
Block 1: Demographic Variables	0.07	
Age		0.19***
Gender (1 = female, 2 = male)		-0.08
Employment status (1 = employed, 2 = unemployed)		0.19***
Source (1 = internet, 2 = paper)		0.01
Block 2: Theory of Planned Behaviour Variables	0.51	
Attitude		-0.47****
Subjective norm		-0.22****
Perceived control		-0.19***
Self-efficacy		-0.06
Block 3: Extended Theory of Planned Behaviour Variables	0.56	
Anticipated regret		-0.19***
Last year, did you have a vaccination for ordinary seasonal flu?, (1 = yes)		-0.04
Do you intend to have a vaccination for ordinary seasonal flu this year? (1 = yes)		-0.14*
Knowledge		0.02
Block 4: Health Belief Model Variables	0.60	
Perceived susceptibility		0.01
Perceived severity		-0.06
*Perceived benefits:*		
Vaccination is a good idea because I feel less worried about catching swine flu		0.07
Vaccination decreases my chance of getting swine flu or its complications		-0.24***
If I get vaccinated for swine fu, I will decrease the frequency of having to consult my doctor		-0.12**
*Perceived barriers:*		
The side-effects of swine flu vaccination interfere with my usual activities		-0.04
I am scared of needles		0.05
I cannot be bothered to get a swine flu vaccination		0.13***

**** p < .0001, *** p < .005, ** p < .01, * p < .05

The model explained 60% of the variance in intention (adjusted R^2 ^= 0.60). Two demographic variables, employment and age were significant predictors. From the original Theory of Planned Behaviour, attitude, subjective norm and perceived control were significant predictors of intention but self-efficacy was not. From the extended Theory of Planned Behaviour, anticipated regret and intention to have a seasonal vaccine this year were significant predictors, but knowledge and past-related behaviour were not. For the Health Belief Model variables, one perceived barrier, "I cannot be bothered to get a swine flu vaccination" and two perceived benefits: "vaccination decreases my chance of getting swine flu or its complications" and "if I get vaccinated for swine flu, I will decrease the frequency of having to consult my doctor" were significant predictors of intention.

## Discussion

To our knowledge this is the first study that has investigated predictors of having a swine flu vaccination in an adult, non-priority group sample during the 2009 pandemic, just as the influenza vaccination had become available. During the current swine flu pandemic, encouraging vaccination uptake focused on providing information [25]. This is similar to interventions to improve uptake of ordinary seasonal influenza vaccination [29], which have met with limited success. However, the current study demonstrated that an extended Theory of Planned Behaviour including some Health Belief Model variables was successful in explaining 60% of the variance in intention to have a vaccination, providing a useful framework to base future interventions on for improving uptake of vaccination in a pandemic situation.

The results indicated that the significant predictors of intention were: being employed, being older, having a positive attitude to swine flu vaccination, scoring high on subjective norm, perceived control, and anticipated regret, intending to have a seasonal flu vaccination this year, scoring low on not being bothered to have a vaccination and believing that swine flu vaccination decreases the likelihood of getting swine flu or its complications and would result in a decrease in the frequency of consulting their doctor.

The original Theory of Planned Behaviour explained 44% of variance, with attitude and subjective norm being significant predictors. However, although perceived control was a significant predictor of intention, self-efficacy was not. Although previous studies have found that self-efficacy is usually a significant predictor of intention and behaviour [30-32], it is not surprising that it was not in this case as during this phase of vaccination in the UK the ease of accessing vaccination was unclear. More surprising, is that perceived control, which is often not associated with intention, [30-32] was a significant predictor of intention to vaccinate. These findings provide further evidence of the differences self-efficacy and perceived control in predicting behavioural intentions.

The extended Theory of Planned Behaviour including some Health Belief Model variables explained another 16% of intention variance. Consistent with previous research [13-15], anticipated regret was a significant predictor of intention to vaccinate. This suggests that an intervention to improve vaccination uptake should use the message that it is better to have the vaccine than regret it later.

Unlike previous studies, past behaviour was not a significant predictor of vaccination intention. However, we measured past behaviour of a related behaviour, having a vaccination for seasonal flu. This suggests that having a vaccine for pandemic influenza is seen as different to seasonal influenza. Yet intention of having a seasonal flu vaccination this year was a significant predictor of intention to have a swine flu vaccine. Recently, evidence has been presented that merely completing measures of intentions from theories such as the Theory of Planned Behaviour can act to encourage the behaviour being investigated [14]. This might have happened in the current study: Just by completing a questionnaire about intention to have a pandemic flu vaccination may have encouraged respondents to intend to have a vaccination for seasonal flu.

In our data, a low score on one perceived barrier: "I cannot be bothered to get a swine flu vaccination" and high scores on two perceived benefits: "vaccination decreases my chance of getting swine flu or its complications" and "if I get vaccinated for swine flu, I will decrease the frequency of having to consult my doctor," were significant predictors of intention. These barriers and benefits could be incorporated into future interventions. Information could be provided about how vaccination can reduce people's worries of contracting pandemic flu and how a vaccination would probably mean fewer visits to the doctor. "Cannot be bothered" was a significant barrier for intending to have a swine flu vaccination, with those in employment particularly likely to give this response. Time factors may of course be important here: to maximise vaccine uptake these vaccinations could be offered near places of work [33]. Unlike in a previous study on health professionals [5], fear of side-effects was not a significant predictor suggesting different concerns between health care workers and the general population. The previous study was of course conducted during the early stages of H1N1, before the vaccine had been properly developed or tested. This may explain the elevated fears of these health professionals about potential side effects.

On the whole, knowledge of swine flu vaccination was not good and this may be linked to the vaccination leaflet: "Swine flu vaccination: what you need to know" [25] being less widely distributed compared to the general information: "Important information about swine flu" leaflet that was distributed to every household in the UK in the early stages of the swine flu alert [24]. Although, knowledge was not a significant predictor of intention, it is important that people do have accurate information about vaccination. It is also important too to address the influence of peers, and the broader news media, in framing understandings of influenza and other such pandemic threats [34,35]. In addition, people may also be more influenced by information obtained from peers and news media than information distributed by the government in print. Such"external" influences also need to be addressed in order to facilitate vaccination uptake.

Although only a minority of respondents filled in their ethnicity, our results were consistent with a survey of 1500 adults in California in 2009, where 65% of African Americans said they did not intend to be vaccinated with the H1N1 vaccine, compared with 14% Whites and 16% Asians [36]. This finding may be related to a historical distrust of the health care and public health system amongst African-Americans [37], as well as anxieties about the safety and effectiveness of influenza vaccination in general [23]. These racial disparities emphasise the need to involve stakeholders in the community and to reassure the community and address their concerns and resistance attitudes and beliefs [23,37]. Future work should examine such racial disparities in beliefs towards pandemic flu in larger samples and with a wider range of ethnic groups.

This study has some limitations. Intention was measured not actual behaviour. However, as the vaccine was not yet available for non-priority groups, this was not possible. The majority of our data were collected online, and while this method is useful for rapid collection of data, and is likely to produce similar results to paper methods [38], it was not a random sample. It should be noted that the online vs. paper version participants did not significantly differ on age, gender and employment status and method of data collection was not a significant predictor of intention. Although snowball sampling methods are commonly used, this non-random methodology may result in a selection bias. Also, as only a minority of respondents completed the question on ethnicity, this variable could not be included in the multiple regression analysis.

## Conclusions

Theoretical frameworks which identify determinants that influence decisions to have a pandemic influenza vaccination are useful. Future studies could use social cognition models to identify predictors of actual vaccine uptake, and potentially compare these findings to predictors of people's intentions to be vaccinated. Once identified, these factors could be used to craft targeted interventions aimed at increasing vaccine uptake.

## Competing interests

The authors declare that they have no competing interests.

## Authors' contributions

LM participated in the design of the study/survey, collected and analysed data, drafted the manuscript. RG participated in the design of the study/survey, assisted with the analysis and helped to draft the manuscript. Both authors read and approved the final manuscript.

## Pre-publication history

The pre-publication history for this paper can be accessed here:

http://www.biomedcentral.com/1471-2458/11/15/prepub
